# Recurrent acute renal failure

**DOI:** 10.4103/0971-4065.73444

**Published:** 2010-10

**Authors:** S. Satish, R. Rajesh, G. Kurian, N. V. Seethalekshmi, M. Unni, V. N. Unni

**Affiliations:** Department of Nephrology, Amrita Institute of Medical Sciences, Kochi, India; 1 Department of Pathology, Amrita Institute of Medical Sciences, Kochi, India; 2 Department of Hematology, Amrita Institute of Medical Sciences, Kochi, India

**Keywords:** Acute tubular necrosis, paroxysmal nocturnal hemoglobinuria, recurrent acute renal failure

## Abstract

While acute renal failure secondary to intravascular hemolysis is well described in hemolytic anemias, recurrent acute renal failure as the presenting manifestation of a hemolytic anemia is rare. We report a patient with recurrent acute renal failure who was found to have paroxysmal nocturnal hemoglobinuria (PNH), on evaluation.

## Introduction

PNH is a rare disorder characterized by intravascular hemolysis. It results from an acquired defect in the erythrocyte membrane resulting in a deficiency of complement defense proteins on the RBC surface. This leads to abnormal susceptibility to complement mediated red blood cell destruction, manifesting as paroxysmal hemolysis.

## Case Report

A 16-year-old boy was admitted to our center with history of passing dark colored urine and reduction in urine output since five days, preceded by fever for two days. There was no history of abdominal pain, skin rashes, joint pains or intake of any medications. He gave history of a similar episode two years back, which was not evaluated. On examination, he had pallor, no edema. BP was 130/80 mmHg and systemic examination was unremarkable.

**Figure 1:**
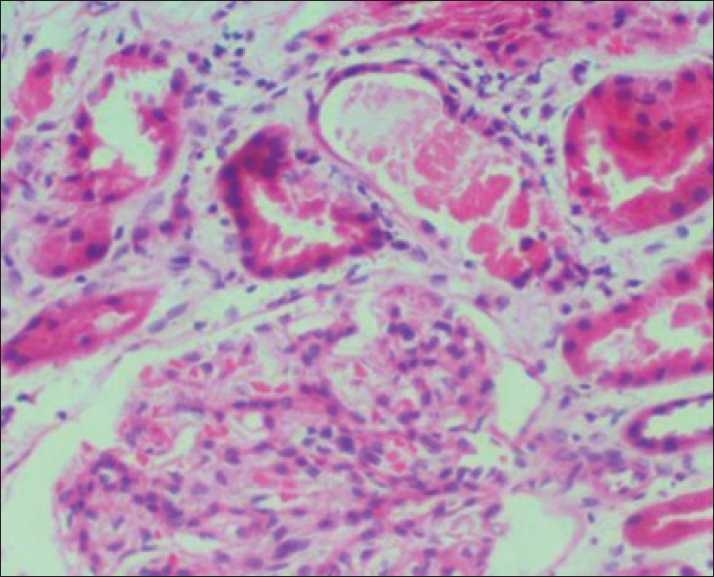
Kidney biopsy :Light microscopy showing features of acute tubular necrosis with intratubular pigment casts (H and E, ×400)

Urinalysis showed 1 + protein, blood 2 + by dipstick, no RBCs on microscopy, Hb 9 g%, total leukocyte count 12,000/mm^3^, platelet count 3,00,000/mm^3^. Serum creatinine 3.2 mg%. Total bilirubin was 3 mg%, indirect 2.0 mg%. AST/ALT/Alkaline phosphatase and serum albumin were normal. Chest x-ray and ultrasonogram of abdomen were normal. ANA and ASO titre were negative and urine culture sterile. Serum complement levels were normal. Peripheral smear showed mild to moderate anisopoikilocytosis, scattered spherocytes, schistocytes and polychromasia. The reticulocyte count was 3.3%. Osmotic fragility, Hb electrophoresis and G 6 PD levels were normal. Coombs test was negative. The renal function worsened after admission and the patient remained oliguric. Hence a kidney biopsy was performed. Kidney biopsy showed normal glomeruli, acute tubular necrosis with prominent pigment casts [Figure 1] which stained blue with Prussian blue stain (Perl’s stain) [Figure 2a and 2b]. Immunofluorescence was negative. Renal functions improved with conservative management over the course of next one week and the patient was discharged, with serum creatinine of 1.8 mg%; he did not undergo dialysis.

**Figure 2a:**
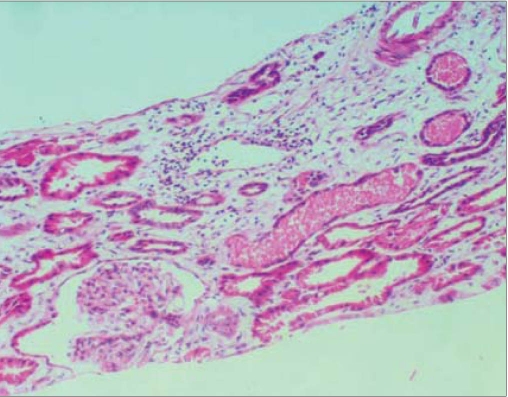
Kidney biopsy:Light microscopy showing prominent intratubular pigment casts (H and E)

**Figure 2b:**
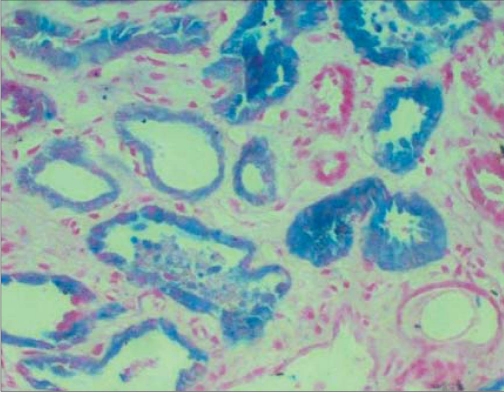
Kidney biopsy: Light microscopy with Perlaes stain (Prussian blue stain) showing uptake of stain by intratubular casts as well as tubular cells

Six months later he presented with a similar episode of fever, followed by oliguric acute renal failure and intravascular hemolysis. Investigations showed serum Creatinine of 4.2 mg%, anemia (Hb 10.8 g%), reticulocytosis (3.5%), schistocytes in peripheral smear and thrombocytopenia (30,000/cmm). In view of the presence of hemolytic anemia and thrombocytopenia, bone marrow aspiration and biopsy were done which revealed hypocellular marrow with trilineage maturation. Ham’s test (acid hemolysis) and sucrose lysis test were positive. Flow cytometric analysis of peripheral blood granulocytes revealed the presence of a PNH clone. The diagnosis of PNH was confirmed. He improved with conservative management and serum creatinine levels came down to 2.0 mg% at discharge. He did not undergo dialysis. On follow-up, serum creatinine improved to normal. He is currently on our follow up.

## Discussion

PNH is a rare acquired clonal disorder, affecting all the three blood cell lines. It arises from an inactivating somatic mutation in phosphatidylinositol glycan class A (PIG-A) gene on the X chromosome necessary for the biosynthesis of a particular glycophosphatidylinositol (GPI) anchor. This GPI anchor attaches a number of crucial protective proteins to the external membrane surface of blood cells. Its absence results in the absence of these proteins. To date, about 20 proteins have been found missing on the blood cells of patients with PNH.[1] Two of these are complement defence proteins CD 55 (PNH decay accelerating factor-DAF) and CD 59, which block complement activation on the cell surface. Deficiency of the GPI-anchored complement regulatory proteins CD55 and CD59 accounts for intravascular hemolysis which is the primary clinical manifestation of the disease. The two other common manifestations of PNH are venous thrombosis and bone marrow failure. Complement activation indirectly stimulates platelet aggregation accounts for the thrombophilia in PNH. The cause of marrow failure is not clear. Hemoglobinuria is intermittent in most patients and never occurs in some, but hemosiderinuria is usually present. Anemia is highly variable, with hematocrits ranging from <20% to normal. RBC are normocytic and normochromic unless iron deficiency has occurred from chronic iron loss in urine. Since hemolysis is due to abnormal sensitivity of RBC to the lytic action of complement, it manifests when the complement cascade is activated, most often by an infection. The hemolysis is often paroxysmal, but, contrary to the name, need not be strictly nocturnal. Hemoglobinuria subsequent to intravascular hemolysis underlies the occurrence of acute renal failure in PNH.

Hemoglobin induced acute tubular necrosis (ATN) is most commonly encountered after blood transfusion reactions. The mechanisms underlying the impairment of GFR in intravascular hemolysis include intrarenal vasoconstriction, intratubular obstruction and tubular toxicity.[3] Hemoglobin is not markedly nephrotoxic when injected *in vivo*.

The products of hemolysis have been found to induce intrarenal vasoconstriction, probably by scavenging the vasodilator nitric oxide (NO) in the renal microcirculation.[3] At acidic pH, hemoglobin is also a source of ferrihemate, a substance that is a potent inhibitor of tubular transport. Hypovolemia and acidosis have been found to predispose experimental animals and humans to pigment induced ATN. Hemoglobin may also potentially induce tubular injury by stimulating local production of OH^-^. The combination of renal ischemia along with ferrihemate deposition produces acute tubular dysfunction and cell injury. Hemoglobin filtered from the glomerular during an episode of hemoglobinuria is converted to methemoglobin in the acidic milieu of the distal tubule, which leads to its precipitation. The occlusion of the distal tubule by the precipitates causes stasis of urine and allows greater time for endocytosis of hemoglobin from the proximal tubule, and the iron released from this dissociation causes free radical oxidant injury.[4]

Although rare, both acute and chronic kidney involvement have been described in PNH. Urinary tract infections and renal vein thrombosis are also known complications of PNH.[4] Renal manifestations include acute kidney injury secondary to intravascular hemolysis and rarely chronic kidney disease.

It has been suggested that causes other than hemolysis are responsible for renal manifestations in PNH.[4] Inability to concentrate urine has been demonstrated even in patients with normal creatinine levels.[4] On histological examination, hemosiderin deposition in proximal tubules was a common feature in most patients, irrespective of the renal function. Renal microinfarcts and interstitial fibrosis have been proposed as a cause of gradual decline of renal function. MRI has been shown to be a reliable non invasive investigation for the diagnosis of renal cortical hemosiderosis.[4] Hemosiderin contains the ferric form of iron that shortens both T1 and T2 relaxation times. This leads to reversal of the normal MRI image of the kidney, where the renal cortex is slightly more hyperintense than the medulla and in T2 weighted images, the lower intensity persists. In PNH, as compared to other hemolytic anemias, the levels of iron in liver and spleen are normal, unless the patient has received multiple transfusions.

The diagnosis of PNH depends on the demonstration of lysis of RBC after complement activation either by acid (Ham’s test) or a reduction in ionic strength (sucrose lysis test).

Flow cytometric analysis of peripheral blood cells (granulocytes or RBCs) using antibodies directed against GPI anchored proteins (GPI-AP) is the most sensitive and informative assay currently available for diagnosis of PNH.[5]

Treatment comprises transfusion therapy in the acute phase and correction of concomitant iron deficiency. Corticosteroids may have a role in attenuating acute hemolytic exacerbations. A humanized monoclonal antibody against complement C5 (eculizumab) has been approved recently for PNH.[2] This prevents activation of C5 and further downstream complement activation and RBC lysis. Thrombosis has to be treated along conventional lines, with thrombolysis in the acute setting and anticoagulation in the long term. Stem cell transplantation has been tried in a few patients who developed marrow failure.

PNH presenting as recurrent acute renal failure is extremely rare. This case has been reported to highlight a rare, but potentially reversible cause of recurrent acute renal failure.
